# Nickel: Human Health and Environmental Toxicology

**DOI:** 10.3390/ijerph17030679

**Published:** 2020-01-21

**Authors:** Giuseppe Genchi, Alessia Carocci, Graziantonio Lauria, Maria Stefania Sinicropi, Alessia Catalano

**Affiliations:** 1 Dipartimento di Farmacia e Scienze della Salute e della Nutrizione, Università della Calabria, 87036 Arcavacata di Rende (Cosenza), Italy; giuseppe.genchi@unical.it (G.G.); graziantonio.lauria@unical.it (G.L.); 2 Dipartimento di Farmacia-Scienze del Farmaco, Università degli Studi di Bari “A. Moro”, 70125 Bari, Italy; alessia.catalano@uniba.it

**Keywords:** nickel, nickel toxicity, nickel allergy, epigenetics, apoptosis, nickel phytoremediation

## Abstract

Nickel is a transition element extensively distributed in the environment, air, water, and soil. It may derive from natural sources and anthropogenic activity. Although nickel is ubiquitous in the environment, its functional role as a trace element for animals and human beings has not been yet recognized. Environmental pollution from nickel may be due to industry, the use of liquid and solid fuels, as well as municipal and industrial waste. Nickel contact can cause a variety of side effects on human health, such as allergy, cardiovascular and kidney diseases, lung fibrosis, lung and nasal cancer. Although the molecular mechanisms of nickel-induced toxicity are not yet clear, mitochondrial dysfunctions and oxidative stress are thought to have a primary and crucial role in the toxicity of this metal. Recently, researchers, trying to characterize the capability of nickel to induce cancer, have found out that epigenetic alterations induced by nickel exposure can perturb the genome. The purpose of this review is to describe the chemical features of nickel in human beings and the mechanisms of its toxicity. Furthermore, the attention is focused on strategies to remove nickel from the environment, such as phytoremediation and phytomining.

## 1. Introduction

Nickel is a hard, ductile, silvery-white transition metal; it is the 28th element in the periodic table. It may exist in several oxidative states (from −1 to +4); nevertheless, the +2 oxidation state (Ni^2+^) is the most widespread in the environment and biological systems [1]. Nickel belongs to the ferromagnetic elements, and it is naturally present in the Earth crust usually in combination with oxygen and sulfur as oxides and sulfides. In combination with other elements, nickel may be present in the soil, meteorites and emitted from volcanoes. About eight billion tons of nickel are in the sea. Thanks to its unique physical and chemical properties, nickel is used in modern metallurgies in a broad variety of metallurgical processes, such as alloy production, electroplating, in the production of nickel-cadmium batteries and as a catalyst in chemical and food industry. The high spread of products containing this metal unavoidably leads to pollution of the environment by nickel and its secondary products at all stages of manufacturing, recycling and disposal. Even though no existing evidence denotes the nutritional value of Ni in humans, it has been recognized as an essential nutrient for some microorganisms, plants, and animal species [2]. Enzymes or cofactors containing nickel are not known in higher organisms, but nickel-based enzymes are well known in the Archaea, bacteria, algae, primitive eukaryotes and plants [3,4,5,6,7]. Nickel is essential in proper growth and development of the plants and has vital roles in a wide range of morphological and physiological functions, such as germination seeds and productivity. However, at high levels nickel alters the metabolic activities of the plants inhibiting enzymatic activity, photosynthetic electron transport and chlorophyll biosynthesis [8]. At the present time, several nickel enzymes have been ascertained, including urease, methyl-coenzyme M reductase, CO-dehydrogenase, Ni-superoxide dismutase, glyoxalase, acireductone dioxygenase, lactate racemase, prolyl cis-trans isomerase and [NiFe] hydrogenase [9,10]. Furthermore, other nickel-dependent enzymes, such as glycerol-1-phosphate dehydrogenase from *Bacillus subtilis* and quercitinase from *Streptomyces* sp. FLA are known [11,12]. Nickel enzymes implicate the use and/or production of gases (CO, CO_2_, CH_4_, H_2_, NH_3_ and O_2_) which play important roles in the global carbon, nitrogen and oxygen cycles [5,13]. The catalytic center of nickel-dependent enzymes is typically coordinated by histidine and cysteine residues with the contributions from aspartate and glutamate [3].

Depending on the dose and length of exposure, as an immunotoxic and carcinogen agent, Ni can cause a variety of health effects, such as contact dermatitis, cardiovascular disease, asthma, lung fibrosis, and respiratory tract cancer [14]. Inhalation exposure in occupational contexts is a main route for nickel-induced toxicity in the respiratory tract, in the lung, and immune system. Inhalation exposure may also affect non-occupationally exposed individuals, mainly those who handle stainless steel and nickel-plated articles, with a high prevalence of allergic contact dermatitis [15,16]. However, the exposure of human beings mainly concerns oral ingestion through water and food as nickel may be a contaminant in drinking water and/or food (Table 1) [17]. Although the molecular mechanisms of nickel-induced neurotoxicity are not yet clear, an important role is due to oxidative stress and mitochondrial dysfunctions. Nickel-induced mitochondrial damage can occur, due to impairment of mitochondrial membrane potential, reduction of mitochondrial ATP concentration and destruction of mitochondrial DNA. According to Song and collaborators [2], the use of antioxidant molecules, such as L-carnitine, taurine and melatonin, molecules that stimulate and amplify antioxidant enzyme activity, can prevent nickel-induced neurotoxicity and carcinogenicity [18,19]. Mitochondrial dysfunctions can interfere with electron respiratory chain and can increase ROS. These three antioxidants, synthesized from all mammals, play significant roles in neurotransmission, detoxification and mitochondrial energy homeostasis, reducing oxidative stress and ROS production [20].

## 2. Chemical Form, Properties and Sources of Nickel Compounds

Nickel (Ni; atomic number 28, atomic weight 58.6934; density 8908 kg/m^3^; melting point 1455 °C; boiling point 2913 °C; electronic configuration [Ar] 3d^8^4s^2^) belongs to group 10 of periodic table along with iron, cobalt, palladium, platinum and five other elements. Nickel is the 24th most abundant element in the Earth’s crust, and it is the 5th most abundant element regarding weight after iron, oxygen, magnesium and silicon. Most nickel on Earth is inaccessible because it is placed in the planet iron-nickel alloy molten outer core, 10% of which is represented by nickel and that lies above Earth solid inner core and below the mantel. In nature, nickel is found in combination with antimony, arsenic, and sulfur; elemental nickel is a silver-white solid metal with high thermal and electrical conductivity. Nickel exists in +2 (Ni^2+^) oxidation state; other valences (+3 and +4) may be found, even if they are less frequent. Nickel is a naturally occurring element that exists in diverse mineral forms [21]. It is resistant to air, water and alkali corrosion, but it is readily soluble at pH < 6.5 in dilute oxidizing acids. Nickel salts of strong acids (chloride, nitrate and sulphate) and organic salts are easily water-soluble, whereas, nickel salts of weak inorganic acids, nickel sulfides and nickel oxides are poorly water-soluble [22]. Compounds of great commercial importance are nickel chloride, carbonate, nitrate, sulphate, acetate, hydroxide and oxide.

Nickel and nickel compounds have many industrial and commercial uses thanks to the chemical properties, gloss and low price. Nickel is used in a wide range of application because of its peculiar combination of outstanding physicochemical properties. It is resistant to very high temperatures, corrosion and oxidation; in addition, it is very ductile, it alloys readily and is fully recyclable. Nickel is used in inexpensive jewelry, keys, paper clips, clothing fasteners (such as zippers, snap buttons and belt buckles), stainless steel household utensils, electrical equipment, armaments, coins, alloy, metallurgical and food processing industries, pigments and catalysts [23,24]. Nickel Raney (in powder or in granular form) is a catalyst employed in reduction reactions, such as the hydrogenation of unsaturated compounds. Nickel alloys are present in solders, surgical steel instruments (5–20% Ni), white gold (10–15% Ni), German silver (10–15%Ni), and sterling silver [22]. Nickel compounds are used in electroplating [25], electroforming and production of nickel-cadmium batteries and electronic equipment. Nickel alloys, such as stainless steel, are largely used in the production of tools, machinery, armaments, aerospace equipment, coins, inexpensive jewelry, medical prostheses and orthodontic materials [26]. Nickel is extensively distributed in the environment as it derives from the anthropogenic activity and natural sources. Nickel released from anthropogenic sources is emitted as oxides, sulfides, soluble compounds, and to a lesser content, as metallic nickel [16]. The greatest presence of nickel compounds in air derives from the combustion of fossil fuels. Direct leaching from rocks and sediments produces high concentrations of nickel in water, where it is present in divalent nickel form, as well as suspended insoluble particles.

Natural sources of atmospheric nickel include wind-blown dust, derived from weathering of rocks and soils, forest fires and volcano activities. The presence of nickel in the air also derives from the combustion of coal, diesel oil and fuel oil, and the incineration of waste and sewage [27]. Other environmental sources of nickel include stainless steel kitchen utensils, inexpensive jewelry and tobacco smoking. It has been demonstrated that each cigarette contains a nickel amount of 1.1 to 3.1 μg; nickel in tobacco smoke may be present as nickel carbonyl, which is extremely hazardous to human health [23]. Another source of nickel exposure in the human population is through dietary exposure; in fact, some vegetables (spinach, asparagus, carrots, broccoli and green beans, tomato), cocoa, chocolate and nuts contain high amounts of this toxic metal [28,29]. Nickel is also involuntarily added to the diet through food processing using stainless steel equipment or through hand to mouth contact [30].

Occupational exposure of several million workers worldwide has given rise to high levels of nickel in blood, urine and body tissues, especially in lung. In this case, workers are exposed to fumes and dusts containing nickel and its compounds; thus, inhalation may be considered the main route of uptake. It is also noted that kitchen kettles may release nickel into drinking water when it is boiled in kettles with nickel-plated elements. Finally, nickel nanoparticles (NiNPs) are among the most widely used nanomaterials, which are employed in many fields, such as catalysts, magnetic materials, biological medicines, conductive pastes and additive to lubricant [31,32,33].

## 3. Nickel Toxicity and Carcinogenicity

In order of nickel abundance in the Earth crust, humans are constantly exposed to nickel. Due to its abundance, natural nickel deficiency does not easily occur; moreover, a nickel-deficient diet is difficult to maintain because of its abundance in food [28]. Human exposure to highly nickel-polluted environments may cause a variety of pathological effects [34,35]. Accumulation of nickel and nickel compounds in the body through chronic exposure may be responsible for a variety of adverse effects on the health of human beings, such as lung fibrosis, kidney and cardiovascular diseases and cancer of the respiratory tract [36,37]. High incidence of nasal and lung cancer in workers exposed to nickel and nickel compounds was observed [37,38,39,40,41,42]. A small fraction of nickel is dermally absorbed, and Ni^2+^ ions and nickel particles penetrate the skin at sweat ducts and hair follicles. Moreover, dermal absorption of this metal is affected by solubilizing agents, such as detergents, and clothes and gloves that behave as a barrier to the skin.

Nickel nanoparticles are associated with reproductive toxicity. Kong et al. [31] exposed female rats by oral gavage to nickel nanoparticles, selecting indicators like reactive oxygen species (ROS), oxidant and antioxidant enzymes, and cell apoptosis-related factors. Nickel nanoparticles decreased the activity of superoxide dismutase (SOD) and catalase (CAT) significantly, and increased the levels of ROS, malondialdehyde (MDA, lipid peroxidation marker) and nitric oxide (NO), in comparison with control groups. Moreover, the authors showed mitochondrial swelling and disappearance of cristae of mitochondrial ovaries in the nickel nanoparticles exposure groups. The mRNA expressions of caspases (cysteine proteases), and the expressions of Cyt C, Bax and Bid proteins on the ovaries significantly increased, while the expressions of B-cell lymphoma-2 (Bcl-2) protein were drastically decreased. Exposure of workers to industrial and research laboratories could be a cause for concern during the production and handling of nickel nanoparticles. Among other causes, even high temperature processes, such as welding, can generate nanoparticles. The results of many in vivo and in vitro researches suggest that nickel and nickel oxide nanoparticles are responsible for lung toxicity, inflammation, oxidative stress and apoptosis [43,44,45].

IARC (The International Agency for Research on Cancer) classified soluble and insoluble nickel compounds as Group 1 (carcinogen to humans), and nickel and alloys as Group 2B (possibly carcinogenic to humans) [46]. The toxic and carcinogenic effects of nickel are related to the way of assumption into the organism. Potential toxicity of nickel and nickel compounds is dependent on their physico-chemical characteristics, as well as the amount, duration of contact and route of exposure. Nickel can enter the body via inhalation, ingestion with food and dermal absorption [47]; however, the route for this element to enter cells is determined by its chemical form. The riskiest route of exposure to nickel is by inhalation [48]. The absorption of nickel particles deposed in the alveolar, tracheobronchial and nasopharyngeal regions of the respiratory system depends on various factors, first of all on the diameter of the inhaled particles, and therefore, the solubility, the quantity deposited, ventilation rate and retention rates [49]. Only particles with a diameter fewer than 100 μm can be inhaled to settle along the respiratory tract. Particles with a diameter of less than 4 μm are deposited in the lower alveolar region of the respiratory system; particles deposited in the tracheobronchial region are between 4 and 10 μm in size, and finally, particles with a diameter between 10 and 100 μm are deposited in the nasopharyngeal region [50]. Water-soluble nickel compounds are absorbed by lungs and removed by the kidneys. They can cause nose and sinuses irritation and may also lead to losing the sense of smell and to the nasal septum perforation. Insoluble nickel compounds remain in the lungs for a longer time, and they are the forms of nickel responsible for cancer. Epidemiological studies have demonstrated increased mortality from lung cancer and from cancer of nasal cavities in nickel refinery workers, because of their chronical exposition to nickel-containing dusts and fumes [37]. Insoluble nickel sulfide (Ni_2_S_3_) is a carcinogen agent for the respiratory tract: When it is inhaled, particles of nickel sulfide accommodate themselves in the lungs of human beings, where they remain in contact with epithelial cells. These nickel particles are removed by macrophages in the digestive tract. Under high exposure to nickel, the macrophage activity of removal could be perturbed, and Ni_2_S_3_ particles may be taken into epithelial cells by endocytosis. In this way, nickel particles are delivered to the nucleus of lung epithelial cells, causing a heritable change in chromosomes, inducing lesions of both double- and single-stranded DNA in cultured human cells (Raji and HeLa cells) [51].

Chen and co-authors, in their study, propose that the enzyme iron- and 2-oxoglutarate-dependent dioxygenases are an important target that mediates the toxicity and carcinogenicity of nickel. The structural motif of this dioxygenase family is a triad of 2-histidine-1-carboxylate that coordinates the Fe^2+^ ion in the catalytic site. According to their hypothesis, two different classes of enzymes in this iron- and 2-oxoglutarate-dependent dioxygenase family, including JMJD1A histone demethylase and DNA repair enzyme ABH2, are all sensitive to inhibition of nickel ions. Their studies with X-ray spectroscopy suggest that nickel is the cause of both JMJD1A and ABH2 inhibition because it replaces iron in their catalytic sites [52,53].

In literature, metallopeptidase with Zn^2+^, Cu^2+^ and Co^2+^ at the active site and sensitive to nickel are known. The *Trypanosoma cruzi* metallocarboxylase (T*c*mcp-1) is the causative agent of the tropical parasitic American trypanosomiasis Chagas disease. This Zn-carboxypeptidase cleaves the C-terminal aminoacid residue from peptides and proteins and lost 54% of its activity upon treatment with 10 μM Ni^2+^. In the active center of this enzyme, there are two histidines and one glutamate residues to bind Zn^2+^ ion. The enzyme nitrous oxide reductase from *Rhodobacter sphaeroides f. sp. denitrificans* catalyzes the reduction of nitrate or nitrite to N_2_ gas under anaerobic conditions. This enzyme contains 4 Cu^2+^, 2 Zn^2+^ and 1 Ni^2+^ atoms per enzyme. Zn and Ni ions cause at 100 μM concentrations reductions in the activity of 100% and 60%, respectively. Hydrogenobyrinic acid a,c-diamide is the substrate of cobaltochelatase, that in *Pseudomonas denitrificans* (Gram-negative aerobic bacterium) catalyzes cobalt insertion in the corrin ring during the biosynthesis of coenzyme B12. Cobaltochelatase is a complex enzyme composed of two different components of Mr 140,000 and 450,000. Each component is inactive by itself, but cobaltochelatase activity is reconstituted upon mixing the two different components. This enzyme is ATP-dependent, and its activity is blocked by nickel [54].

It is known that nickel inhibits many enzymes that do not need metal cations to carry out the catalysis. This inhibition takes place when the nickel binds to particular amino acids in the active site of the enzyme, such as cysteine, histidine, glutamate and lysine, blocking the catalytic activity, or binds to secondary sites of the enzyme influencing allosterically its activity. However, in most cases, the inhibition mechanism is not known.

ATP: Cob (I) alamin adenosyltransferase from *Salmonella enterica* catalyzes the final step in the conversion of vitamin B12 to coenzyme B12, namely, the adenylation of cobalamin/vitamin B12 to adenosylcobalamin/coenzyme B12. This enzyme, that contains iron in its active site, is inhibited upon exposure to 100 μM Ni^2+^, losing about 50% of its activity. At nickel concentration higher than 100 μM, the activity of this enzyme did not decrease below 50%. This result suggests that nickel binds to an allosteric site, rather than displacing iron from the catalytic center [55,56,57].

Nickel at a concentration as low as 100 nM inhibits the activity of the enzyme N-carbamoyl D-amino acid amidohydrolase from *Agrobacterium radiobacter*. This enzyme, which has the triad of Cys/Glu/Lys amino acids in the active site, as well as three histidines, is important for the production of the β-lactam antibiotic. [58].

Schaeffer and co-authors have shown that the treatment of the pyroglutamyl peptidase I (PPI) enzyme from *Leishmania major* to 100 μM Ni^2+^ causes the loss of 48–50% of its activity. This enzyme, which belongs to the cysteine peptidase family, hydrolyzes the N-terminal L-pyroglutamate residues that play an important role in protein metabolism and defense against antibiotic peptides. The *Leishmania major* PPI active site catalytic triad of Glu101, Cys210, and His234 [59].

## 4. Nickel Allergy

Metals, such as gold, silver, nickel, titanium, chromium and copper, are ubiquitous in our environment and are widely used in costume jewelry, coins, mobile phone and orthodontic materials [28,60,61,62]. The orthodontic patients are exposed to a considerable amount of nickel, cobalt, titanium and other metals deriving from alloys. The microbiologic and aqueous oral environment combined with pH of saliva, intake of drinks, food and mouthwashes facilitate corrosion resulting in the release of ions from orthodontic appliances into oral tissues and saliva of patients. These ions released from orthodontic appliances cause contact dermatitis, hypersensitivity, cytotoxicity and DNA damage [63,64,65]. Among these metals, nickel is the most frequent cause of metal allergy. Clinically, nickel allergy sometimes occurs when nickel-containing items are in direct and prolonged contact with the skin, leading to corrosion of nickel by sweat, releasing nickel ions to be absorbed through the skin and initiating an allergenic effect. Once sensitized, individuals can develop contact dermatitis, lichen planus, dyshidrotic eczema, labial desquamation, angular chelitis, periodontitis, stomatitis with mild to severe erythema, loss of taste and numbness [66]. A particular problem is due to the use of nickel for coinage [67,68], such as the European one and two euro coins [69,70]. The EU Nickel Directive in agreement with European Chemical Agency (ECHA) poses limits on the amount of nickel that may be released from jewelry and other products destined to come to direct and prolonged contact with the skin. These limits are known as migration limits—(a) 0.2 µg/cm^2^/week for post assemblies which are inserted into pierced ears and other pierced parts of the human body; (b) 0.5 µg/cm^2^/week for other products intended to come into direct and prolonged contact with the skin. The quantitative test for nickel ion release is the European Standard EN1811, which consists of placing an object in an artificial sweat solution for one week, then nickel release is measured by atomic absorption spectroscopy or any other technique as inductively coupled plasma mass spectrometry (ICP-MS). Wear and corrosion can be simulated by a method known as EN 12472.

By the early 1930–1935 observations of nickel dermatitis produced by objects in everyday use were reported by Rothman and Preininger about the use of coins, by McAlester AW and McAlester AWJr (1931) about the use of spectacle frames and by Du Bois about the use of wrist watch [71,72,73,74].

Literature dates indicate that women are more prone to dermatitis than men. In fact, approximately 13–18% of females and 3–6% of males are allergic to nickel [75], and this may be due to greater contact with nickel-containing items, such as jewelry, buttons, certain shampoos and detergents, and pigments [76]. According to dermatologists, the frequency of nickel allergy is still increasing, and it can be explained by the fashionable piercing, and nickel-containing devices used in medicine like coronary stents and endo prosthesis [38]. Nickel is also a component of many orthodontic materials, as a nickel-titanium alloy with a concentration of about 50%. It is also present in stainless steel in archwires and brackets, with a concentration of approximately 8%. It is also present in surgical stainless steel instruments (10–20% concentration without release nickel at a rate of more than 0.2 µg/cm^2^/week). If symptoms are present with a nickel hypersensitivity diagnosis, the nickel-titanium apparatus possibly present should be removed and replaced with a stainless steel device and preferably with a titanium-molybdenum alloy, which does not contain nickel [77]. Saliva, some foods and oral hygiene products containing fluoride potentially corrode and solubilize nickel in the alloys, releasing nickel ions onto the oral mucosa [78].

Approximately 10–15% of the population in the Earth suffers from nickel allergy, and many are unable to wear jewelry or handle coins and other objects that contain nickel. Many agents have been developed to reduce the penetration of nickel through the skin, but few formulations are safe and effective. In 2011, Vemula and co-authors showed that the penetration of nickel ions into the skin could be prevented, by applying a thin layer of glycerin containing nanoparticles of calcium carbonate or calcium phosphate either in vitro on an isolated piece of pig skin or in vivo on the skin of mice. The nanoparticles may capture nickel ions by cation exchange, and remain on the surface of the skin, allowing them to be removed by simple washing with water. Therefore, the use of nanoparticles with diameters smaller than 500 nm in topical creams may be effective in limiting the exposure to metal ions that can cause skin irritation [79].

Prevention strategies could reduce the awareness of persons that suffer from allergic contact dermatitis. In a clinical immunology review, it is hypothesized that prevention of exposure to nickel could diminish the number of those that are sensitive to nickel by one-quarter to one-third. Therefore, the identification of sources of nickel is vital to understanding the nickel sensitization processes. Food items like chocolate and many other products, such as zippers, buttons, cell phones, orthodontic braces and eyeglass frames may contain nickel. For example, objects with sentimental value (heirlooms, wedding rings) could be treated with an enamel or rhodium plating [80].

Moreover, nickel-containing cobalt and titanium alloys have become ubiquitous in the manufacturing of neurovascular medical devices. In fact, aneurysm clips, endovascular devices and stents containing varying proportions of nickel (14–35%) are used in treatments of cerebral aneurysms [81]. Allergic reactions to nickel have been implicated in complications related to coronary stents, including migraine headaches, fever, dyspnea, dermatitis and pericarditis [82].

Nickel released from various alloys is a potent allergen or hapten that can trigger skin inflammation. Nickel penetrates the skin and activates epithelial cells, producing cytokines or chemokines. The reaction involves the activation of antigen-presenting cells and T cells in complex immune responses. Activated antigen-presenting cells migrate to the lymph nodes where nickel present the allergen or hapten to naive CD4+ T cells. Following the re-exposure to the same allergen or hapten, these T cells become stimulated and duplicate themselves. Subsequently, they enter in the bloodstream and produce visible signs of hypersensitivity 48–72 h after allergen exposure. After repeated exposures to nickel, the clones of T-cells reach “threshold” value, and the skin develops a rash that can manifest as acute, subacute, or chronic eczema-like skin patches. The skin reaction can take place at the site of contact, or sometimes diffuse to the rest of the body. Cutaneous exposure can cause localized erythematous, pruritic, vesicular, and scaly patches. Ingestion of food containing nickel or nickel compounds may cause adverse systemic reactions [83]. However, the pathogenesis and mechanisms of the allergic response are highly complex, and the precise mechanisms of nickel allergy remain still not completely clarified [66].

## 5. Epigenetic Effects of Nickel

Epigenetics refers to heritable modifications in gene expression that do not involve a change in the nucleotide DNA sequence. The principal molecular mechanisms mediating epigenetic regulation of gene expression are DNA methylation [84], histone modification and microRNA expression, each of which alters how genes expressed without altering the underlying DNA sequence. These processes may be influenced by a variety of environmental factors, and their dysregulations are implicated in many diseases states [85,86,87].

DNA covalent methylation involves the covalent addition of a methyl group to the cytosine to form 5-methylcytosine in the presence of the enzyme DNA methyltransferase with SAM (S-adenosylmethionine) as methyl group donor. In mammalian cells, the majority of 5-methylcytosine is located in the contest of CpG (CytosinepGuanine) dinucleotides to form the so-called CpG islands; in mammalian genome, the length of a CpG island is generally between 300 and 3000 base pairs. In mammals, 70–80% of CpG cytosine are methylated [88]. DNA methylation is involved in the regulation of many cellular processes, including chromatin structure and remodeling, genomic imprinting, chromosome stability and gene transcription [89]. Histone modification provides another mechanism of epigenetic regulation. Histones are nuclear proteins that package DNA in nucleosomes, the units of chromatin structure. The N-terminal long tail of H3 and H4 histones [90] are subjected to a variety of post-translational covalent modification reactions, like cytosine and arginine methylation, lysine-acetylation and -citrullination, serine and threonine phosphorylation, ADP-ribosylation, -ubiquitination and -sumoylation that influence chromatin structure and gene expression. MicroRNAs are single-stranded RNAs of approximately 21–23 nucleotides in length that are transcribed from DNA, but not translated into proteins. Mature microRNAs are partially complemental to one or more messenger RNA molecules. MicroRNA main function is to down-regulate gene expression by interfering with mRNA functions [91,92].

Nickel ions are able to induce heterochromatinization by binding to DNA-histone complexes and initiating chromatin condensation. Both water-insoluble nickel sulfide (NiS) and water-soluble nickel sulphate (NiSO_4_) and nickel chloride (NiCl_2_) are human carcinogens, although insoluble nickel compounds are more potent carcinogens than the soluble ones [93]. Nickel compounds can produce histone hyperphosphorylation (H3S10), hypermethylation (H3K4) and hyperubiquitination (H2A and H2B), inducing epigenetic effect that can act on gene expression [94,95,96]. Their carcinogenic potential is thought to be due to the ability to precipitate epigenetic changes [97]. In a study on reconstituted homogeneous oligonucleosomal arrays in the presence of Mg^2+^ or Ni^2+^, Ellen and collaborators observed that nickel condenses chromatin to a greater extent than magnesium, the natural cation present into the cell [98]. Given that chromatin condensation is a necessary step towards heterochromatinization, that is in turn associated with various gene expression-repressive changes, Ellen and co-authors concluded that the carcinogenic effect of nickel might be mediated by modulation of chromatin, such as heterochromatinization. The authors speculated that heterochromatinization could be followed by DNA methylation, and the outcome might be cell transformation, tumor progression and oncogenesis, if these silenced chromatin regions contained tumor suppressor or senescence-related genes.

Salnikow and Zhitkovich found in vivo changes in DNA methylation in nickel-induced tumors [99]. Intramuscular injection of nickel sulfide into wild type *p53^+/−^* mice induced malignant histiocytomas in all mice and this tumor development was associated with hypermethylation of the tumor suppressor gene *p16* in all tumors [100]. Kowara et al. showed that both in vivo and in vitro nickel exposure reduces Fiht protein expression in B200 mouse cell line and in murine sarcomas [101].

Exposure of cells to nickel chloride and nickel sulfide resulted in intracellular nickel accumulation, a loss of acetylation at all four core histones in human lung A549 cells, and an increase of dimethylation of histone H3 lysine 9 (H3K9me2), inducing transgenic silencing [101,102]. Nickel hypermethylation had no effect on histone methyltransferases, but was found to be due to the inhibition of H3K9 demethylase by replacing in the catalytic center the Fe^2+^ by Ni^2+^ [53,103,104]. Nickel chloride and nickel sulfide may also induce both histone ubiquitination and phosphorylation in A459 cells [94,96]. Treatment of A459 cells with soluble or insoluble nickel compounds resulted in increased levels of H2A and H2B ubiquitination in a dose- and time-dependent manner, indicating that both soluble and insoluble nickel compounds share similar epigenetic effects in inducing histone ubiquitination.

Karaczyn et al., using isolated bovine histone H2A, as well as different rodent (CHO, Chinese hamster ovary; NRK-52, rat renal tubular epithelium) and human (HPL1D, human lung epithelium) cell lines, showed that nickel binds to the C-terminal TESHHKAKGK sequence of histone H2A and hydrolyzes the E-S peptide bond, resulting in the release of the C-terminal octapeptide SHHKAKGK from histone H2A [105]. The authors speculated that the truncation of histone H2A may alter the chromatin structure and affect gene expression, which may be involved in mediating nickel toxicity and carcinogenicity.

Nickel exposure has been associated with DNA hypermethylation and transcriptional repression of tumor suppressor genes in vitro and in vivo. In addition, nickel modulates various and different histone post-translational modifications, such as acetylation, methylation, sumoylation, ubiquitination and ADP-ribosylation, by targeting the enzymes that add or remove the specific marks. In addition, nickel may interfere with microRNA network to degrade mRNA or block protein synthesis [88]. Then, piling evidence suggests that epigenetic modifications working in concert with genetic mechanisms to regulate transcriptional activity are dysregulated in many diseases, including cancer. Aberrant DNA methylation, histone modifications, are key epigenetic mechanisms associated with tumor initiation, cancer progression, and metastasis.

## 6. Teratogenicity of Nickel Compounds

Teratology studies the congenital malformations, retardation growth and their cause. Particularly in early pregnancy, intrauterine exposure to a toxicant stimulates changes in the embryo and fetus that lead to malformations and stillbirths. The teratogenic agents include rubella virus, protozoal infections. Ionizing radiations, hyperthermia, pharmacological drugs as thalidomide and retinoic acid, corticosteroids, antimalarial and antihypertensive agents, industrial pollutants as toluene and pesticides, heavy metals, such as Hg, Cd and Ni. Even bad mother life behavior like alcohol abuse, cigarettes and narcotics during pregnancy negatively affects the embryo and the fetus. Health problems of the mother, such as diabetes mellitus and rheumatoid arthritis, are added to the list of teratogens. The lack of folate in the diet of pregnant women results in spina bifida in the newborn. Nickel can cross the placenta and has embryo toxic and teratogenic properties.

Sanderman and co-authors [106,107] studied the effects of Ni carbonyl, Ni(CO)_4_, and nickel sulfide (Ni_2_S_3_) administered to pregnant hamsters and female rats. In the first paper, Ni carbonyl was administered to pregnant hamsters on different days of gestation; then the dams were sacrificed on day 15 of gestation, and the fetuses were tested for malformations. Progeny of dams treated with Ni(CO)_4_ (0.06 mg/L/15 min) on days 4 and 5 of gestation included fetuses with cystic lungs, exencephaly, exencephaly plus fused rib and anophthalmia plus cleft palate. In addition, in the progeny of dams treated with Ni carbonyl on days 6 and 7 of gestation, there were two fetuses with hydronephrosis and one with fused ribs. In the second paper, Sunderman illustrated in three experiments the effects of Ni carbonyl and nickel sulfide on the progeny of Fisher rats. Intravenous injection (11 mg Ni/kg) on day 7 of gestation to pregnant dams caused malformations of fetuses, including anophthalmia, microphthalmia and cystic lungs, and fetal mortality. In the second experiment, male rats were treated by inhalation with Ni carbonyl (0.05 mg Ni/L/15 min) two to six weeks to breeding without impair fertilization rates or reproductive yields. However, administration of organic Ni compound by intravenous injection (22 mg Ni/kg) decreased the number of live pups during the fifth week, due to chromosomal damage during the meiosis of spermatogenesis. In the last experiment, female rats were treated by intrarenal injection with Ni_2_S_3_ (30 mg Ni/kg) one week prior to breeding, and the result was an intense erythrocytosis in the dams without cause erythrocytosis in the pups; but instead, pups born from dams treated with Ni_3_S_2_ had decreased hematocrits at two weeks postpartum.

Leonard and collaborators [108,109] with their experimental studies have demonstrated that nickel compounds have potent effects of carcinogenicity and teratogenicity. Apart from an increase of prenatal and natal mortality, Ni may cause a different type of malformation in the embryos. It was hypothesized by Leonard et al. that the prenatal nickel effects could partially be due to the changes in mitosis, leading to cellular death.

The study of Saini et al. [110] was conducted to assess the potentially harmful effect of Ni (NiCl_2_^.^6 H_2_O) on the fetuses of Swiss albino mice. Ni (46.125, 92.25, 184.5 mg Ni/kg body weight) was administered orally from days 6 to 13 of gestation period. On day 18 of gestation, dams were sacrificed, and uteri were examined. After the administration of the three different dosages of Ni, Saini noted a lower number of implant sites and placental weight compared to the respective controls. Saini and co-authors after the nickel treatment of mice have noticed different malformations in fetuses, such as for example hydrocephaly, microphthalmia, exophthalmia, club foot and umbilical hernia. In addition, bone malformations have been highlighted like reduced ossification of nasal, frontal, parietal and supraoccipital bones, reduced fuses sternebrae and caudal vertebrae, and absence of carpal, metacarpal, tarsal, metatarsal and phalanges.

The results of these researchers indicate the vulnerability of the rats and mice fetus to nickel during prenatal exposure.

## 7. Nickel-Induced Apoptosis

Apoptosis is a process of programmed cell death that occurs in all multicellular organisms [111,112], and takes place with biochemical events that lead to characteristic cellular changes that have cell death as the last step. These changes include cell shrinkage, blebbing, nuclear fragmentation, chromatin condensation, chromosomal DNA fragmentation and global RNA decay [113]. The average adult human loses between 50 and 70 billion cells each day, due to apoptosis.

In contrast to necrosis, which is a form of traumatic cell death that is a consequence of acute cellular injury, apoptosis is a highly regulated and controlled process that confers advantages during an organism lifecycle. Unlike necrosis, apoptosis produces cell fragments called apoptotic bodies that phagocytic cells are able to ingest and quickly remove before the content of the cell can split onto surrounding cells and cause damage to the neighboring cells.

Two principal pathways exist by which cells can undergo apoptotic death, known as the intrinsic (also called mitochondrial pathway) and the extrinsic pathways. The former is activated by intracellular signals generated when cells are stressed and are related to the release of Cyt C from the intermembrane space of mitochondria. The latter is activated by extracellular ligands binding to cell-surface death receptors (TNF, Tumor Necrosis Factor-receptor family), which leads to the production of the death-inducing signaling complex. In the intrinsic pathway, the cell kills itself because of cellular stress, while in the extrinsic pathway, the cell kills itself because of signals received from other cells. Both pathways induce cell death by activating caspases (cysteine proteases) or enzymes that degrade proteins.

**Figure 1:**
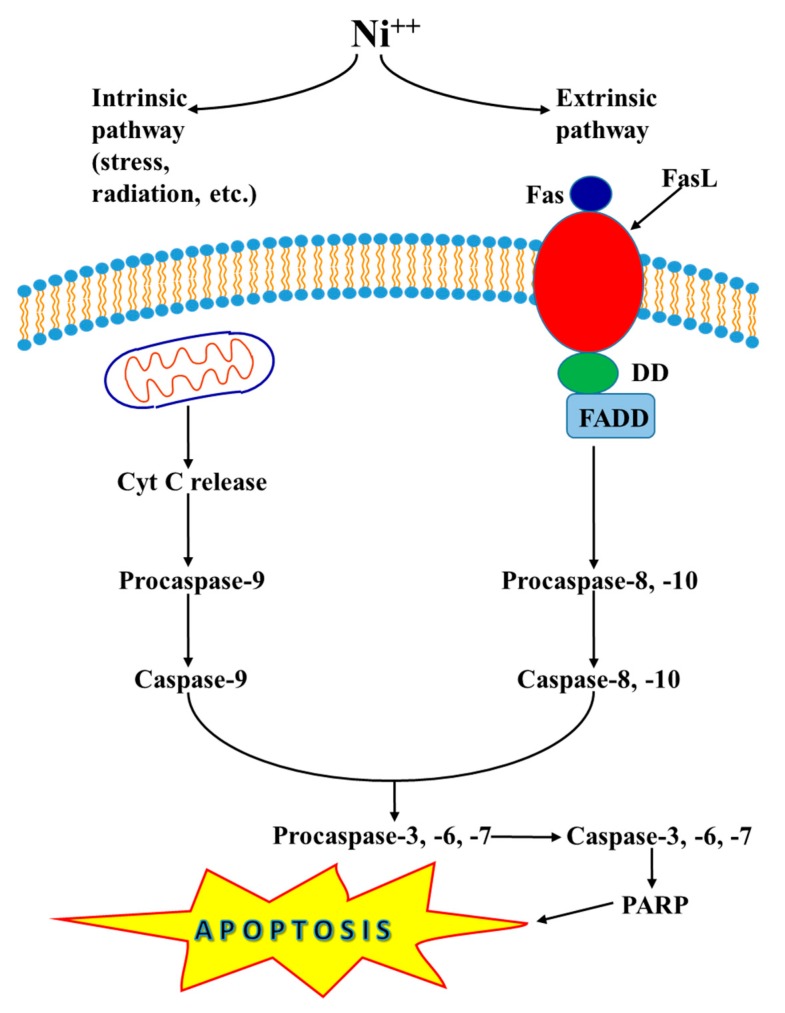
Ni^2+^-induced mitochondria-apoptosis and caspase-dependent apoptosis.

Nickel ions allow the release of Cyt c from mitochondria in the cytosol, where Cyt C cleaves procaspase-9 with activation of caspase-9, which in turn activates caspase-3, -6, and -7. These caspases act on PARP, which induces apoptosis. On the cell surface, the Ni ions promote the interaction between Fas (First apoptotic signal) and FasL (Fas Ligand) with the formation of the death-inducing signaling complex, which contains FADD and procaspase-8 and -10 that are activated to caspase-8 and -10. In the cell caspase-8 and -10 cleave and activate the effectors of proteases, such as caspase-3, -6 and -7 that act on PARP, which lead to apoptosis (Figure 1). Moreover, some members of Bcl-2 family of proteins inhibit apoptosis [114]. In addition to its importance as a biological phenomenon, defective apoptotic processes have been involved in a wide variety of diseases. Excessive cell death is responsible for many neurodegenerative diseases, whereas, failure to undergo apoptosis results in autoimmune diseases and uncontrolled cell proliferation, such as cancer.

Su and co-authors reported that NiSO_4_ induces DNA damage, apoptosis and oxidative stress in rat testes. This study also explored the effect of protection of grape seed proanthocyanidin extract (GSPE) against nickel toxicity in the testes. The authors treated rats with normal saline, nickel alone (1.25, 2.5, and 5.0 mg/kg/day), and nickel (1.25, 2.5, and 5.0 mg/kg/day) in the presence of GSPE (50 and 100 mg/kg/day). After a period of 30 days treatment, nickel (2.5 and 5.0 concentrations) exhibits reproductive toxicity by decreasing sperm motility, while GSPE enhances sperm motility [115].

Zou and collaborators reported that nickel sulphate induced apoptosis in rat testicular Leydig cells via activating ROS-dependent mitochondria [116]. The results of the study showed that nickel sulphate induced ROS generation with nucleus deformation and apoptosis in Leydig cells, which were attenuated by ROS inhibitors of NAC (N-acetylcysteine) and TEMPO (2,2,6,6-tetramethyl-1-piperidinyloxy). In vitro studies on nickel-induced apoptosis suggested that nickel compounds can promote apoptosis in human epatoma cells [117], keratinocytes [118], human T hybridoma cells [119], human breast cancer (MCF-7 cells), abrogated by antioxidant curcumin [120], human liver cells (HepG2) [121], as well as human neutrophils [122]. Ma and co-authors demonstrated that nickel nanowires induce apoptosis in HeLa cells through ROS generation and that ROS induce HeLa cells apoptosis through mitochondrial membrane damage or activation of cell cycle checkpoints [123].

Pan and co-authors [124] investigated the effect of Ni-smelting fumes on cell viability, mitochondrial damage and apoptosis in NIH/3T3 cells. Treatment with Ni-smelting fumes increased mitochondrial permeability transition pore opening and decreased mitochondrial activity of the complex I (NADH: Ubiquinone oxidoreductase), complex II (succinate dehydrogenase) and complex IV (cytochrome c oxidase) of the mitochondrial respiratory chain. The Ni-smelting fumes downregulated Bcl-2, procaspase-3 and -9, and upregulated caspase-3, and -9. In mammalian cells, Ni-smelting fumes caused significant cytotoxicity, oxidative stress, mitochondrial damage and apoptosis through the intrinsic pathway.

## 8. Nickel Phytoremediation and Phytomining

Since heavy and transition metals (Hg, Pb, As, Cu, Ni and Cr) are no degradable by microorganisms, they accumulate in the environment (soil, water and air) and subsequently contaminate the food chain, generating a risk to human health. Some heavy and transition metals are carcinogenic, teratogenic and endocrine disruptors; some others cause neurological and neurodegenerative diseases and behavioral changes in human beings [28,60,61,62]. Different physical and chemical methods used for remediation of heavy and transition metal pollution suffer from high cost, alteration of soil properties and disturbance of soil microflora and creation of secondary pollution problems. Phytoremediation is a solar-driven technology that uses plants to clean up soil, air, and water contaminated with hazardous chemicals. It removes pollutants, including toxic and radioactive metals, from the environment by using plants [125]. Phytoremediation is a better solution to pollution, because it is environment-friendly and an aesthetically pleasing with good public acceptance. It is a cost-effective plant-based approach that takes advantage of the ability of plants to concentrate elements and compounds from the environment and to metabolize dangerous molecules in their tissues.

It refers to the natural ability of plants, called hyperaccumulators, to bioaccumulate, degrade or render harmless contaminants in soils, water and air. Plants can be defined as a hyperaccumulator if it can concentrate the pollutants in a minimum percentage which varies according to the pollutant involved (for example, more than 1000 mg/kg of dry weight for nickel, copper, cobalt, chromium or lead; or more than 10,000 mg/kg for zinc or manganese). This capacity for accumulation is due to a hypertolerance or phytotolerance. It is the result of adaptive evolution from the plants to hostile environments over many generations. Contaminants, such as heavy metals, pesticides, solvents, explosives, and crude oil have been eliminated with phytoremediation projects worldwide. Phytoremediation may be applied wherever the soil or static water environment has become polluted or is suffering ongoing chronic pollution.

Techniques of phytoremediation include phytoextraction, phytofiltration, phytostabilization, phytodegradation and phytodesalination. Phytoextraction consists in the contaminants uptake from soil and water by plants roots [126]; phytofiltration is the removal of pollutants from contaminated wastewater by plants [127]; phytostabilization is the use of plants for stabilization of contaminants reducing the mobility of pollutants and preventing they entry in the food chain [128]; phytodegradation is the destruction of organic xenobiotics by plants thanks to enzymes like oxygenase and dehalogenase [129], phytodesalination refers to the use of halophytic plants for removal of sodium chloride from salt-affected soils in order to enable them for supporting normal plant growth [130,131].

The extraction of nickel, cobalt, iron, platinum, palladium and other heavy metals from soil by cropping them with hyper accumulating plants that concentrate these metals in aerial parts of the plants, which are then harvested, dried and smelted, allows to recover the metals in a process known as metal phytomining [132]. There are many Ni hyper accumulators, i.e., plants which accumulate more than 1000 mg Ni/kg of dry weight in their shoots when grown in natural habitats. In late 1948, Minguzzi and Vergnano discovered that *Alyssum bertolonii* Desv. (Brassicaceae) had an extraordinarily high Ni content of about 10 mg/g [l%] in a dried matter which translated to well over 10% of this element in the ash [133].

In 1976, Jaffrè et al. reported that in New Caledonia an endemic tree (*Pycnandra acuminata*, first known as *Sebertia acuminata*) has extraordinary ability to accumulate Ni in its latek of blue-green color, due to the presence of nickel (about 25% of the latek dry weight). New Caledonia is an archipelago with a third of its surface covered by Ni-rich soil of ultramafic rocks. Jaffrè and co-authors consider that the rich New Caledonian flora contains 2145 species adapted to ultramafic soils among which 65 are Ni hyperaccumulators [134].

Analyses of New Caledonia hyperaccumulators revealed that Ni was associated with citrate complex in the *Pycnandra acuminata* [135,136]. Callahan et al. studies of gas chromatography mass spectrometry revealed the presence of Ni-nicotianamine complexes in most of the New Caledonian species, including *Pycnandra acuminata*. In addition to citric acid, a methylated aldaric acid (2,4,5-trihydroxy-3-methoxy-1,6-hexan-dioic acid) appears to be one of the most abundant small organic molecules present in the latex; others Ni complexes were detected, as well as malic, itaconic, galacturonic, tartaric and aconitic acids [137].

The experiments of Giordani et al. [138] studied the phytoremediation of soil polluted by nickel using agricultural crops on seven herbaceous crops as burley (*Hordeum vulgaris*), bean (*Phaseolus vulgaris*), cabbage (*Brassica juncea*), ricinus (*Ricinus communis*), sorghum (*Sorghum vulgare*), spinach (*Spinacea oleracea*) and tomato (*Solanum lycopersicum*). At the end of experiments, leaves, roots, stems, fruits or seeds were separately collected, oven-dried at 100 °C, weighed, milled and analyzed. It was shown that spinach, ricinus and cabbage were able to store nickel in the leaves. The bean, barley and tomato showed a high concentration of nickel in leaves and in stems. The bean was the most efficient in the storage of nickel in fruits or seeds and roots—unlike tomato, sorghum, ricinus and barley, which showed a storage capacity lower than that of a bean. With regard to the removal of nickel, spinach was the most efficient as it contains the highest levels of the metal per gram of dry matter with respect to the other six herbaceous crops [138]. The Ni hyper accumulator *Alyssum murale* has been developed as a commercial crop for phytoremediation/phytomining Ni from metal-enriched soils [139]. Nickel hypertolerance is often specific. It undergoes vacuolar sequestration via epidermal compartmentalization, whereas, cobalt present in the xylem or leaf apoplasm was excreted from leaves and subsequently sequestered on leaf surfaces as soluble precipitate. Therefore, the specialized biochemical processes linked to Ni hypertolerance in *Alyssum murale* did not confer hypertolerance to cobalt. Broadhurst and Chaney [140] co-cropped *Alyssum murale* (Ni hyperaccumulator), *Alyssum montanum* (Ni non-hyperaccumulator), and ryegrass *Lolium perenne* in a natural serpentine soil to affect Ni, Cu, Fe and Mn uptake. After four months with standard inorganic treatment, *A. murale* leaves and stems contained 3600 mg/kg Ni. Moreover, the concentration of Ni and Mn were significantly higher in *A. murale* respect to *A. montanum* or *L. perenne*. Both *Alyssum* species not accumulated Cu, while *L. perenne* accumulated only 10 mg/kg Cu. Besides, co-cropping *A. murale* with *L. perenne* reduced Fe and Mn concentrations in *A. murale* [141]*. Psychotria douarrei* and *Geissois pruinosa* are other plants called hypernickelophores, which accumulate more than 10,000 mg Ni/kg [141]. Fernando et al., using the colorimetric reagent dimethylglyoxime, indicated high levels of Ni in the leaves of *Rinorea niccolifera* (Violaceae) [142,143]. Subsequent chemical analyses of the plant tissues revealed foliar nickel concentrations varying from 7168 to 18,388 mg/kg on a dry weight basis. The data are based on six sets of plant tissue samples of *Rinorea niccolifera* collected from two sites of Luzon Island (Zambales Province, Municipalities of Santa Cruz and Candelaria, Philippines). As this species surpasses the 10,000 mg/kg Ni accumulation level in the leaves, it is regarded as a “hypernickelophore”. The studies of Roccotiello et al. highlight that Mediterranean *Alyssoides utriculata* leaves are Ni-hyper accumulators (higher than 1.0 g/kg) and can be used for Ni-phytoextraction purposes and for cleaning Ni-contaminated areas [144]. In its second paper, Roccotiello et al. [145] investigated in pot experiments the effect of different Ni concentration (0–500 mg Ni/L) on the physiology of *Alyssoides utriculata*. The results showed that the concentration of this transient metal is higher in leaves than in roots, and at the higher concentration tested (500 mg Ni/L), *A. utriculata* accumulates 1.1 g Ni/kg leaves as previously found. Plant water content increases significantly with Ni accumulation without affecting chlorophyll fluorescence parameters. In fact, the photosynthetic efficiency of *A. utriculata* is stable among Ni treatments (always ≥0.8).

The bioaccumulation and distribution of Ni were also elucidated in three plant species: *Phyllanthus balgooyi*, Phyllanthus securinegioides (Phyllanthaceae) and *Rinorea bengalensis* (Violaceae) that occur in Sabah (Malaysia) on the Island of Borneo. This study by Van der Ent et al. showed that Ni is mainly concentrated in the phloem, in roots and stems (up to 16.9% Ni in phloem sap in *Phyllanthus balgooyi*) in all three species [146]. However, regarding their leaves: In *Phyllanthus balgooyi* the highest Ni concentration is in the phloem, but in *Phyllanthus securinegioides* and *Rinorea bengalensis* in the epidermis and in the spongy mesophyll (*Rinorea bengalensis)*. All three species have a highly distinctive tissue Ni distribution patterns with extreme levels of accumulation in the phloem of the root and stem. The phloem tissues in the main stem of all three species are green, due to the exceptionally high concentration of Ni^2+^ ions, and appear to act as a ‘sink’ with Ni concentrations reaching up to 2.1% in *Rinorea bengalensis* and up to 16.9 wt%, in the phloem sap from *Phyllanthus balgooyi.*

Many strategies have been used for solving the problem of heavy metals pollution in the environment. The bioremediation methods for decreasing the amount of heavy metals in the environment have attracted the attention of many researchers. Plants, bacteria, fungi and algae are usually used for bioremediation of heavy metals [147], and in the literature, there are now many publications of biosorption of nickel, among others *Pseudomonas fluorescent* [148], *Bacillus cereus* [149], *Saccharomyces cerevisiae* [150], and filamentous fungi *Trichoderma atroviride* strains F6 [151].

In their studies, Abdel-Monem et al. [152] used *Bacillus subtilis* 117S and *Pseudomonas cepacia* 120 S to remove Ni from bacterial biomass, sludge, tea and sawdust. The authors of this study have shown that the biosorption capacity of nickel by bacterial biomass was greater than that by sludge, tea and sawdust. Moreover, the nickel removal increased with contact time from 1 to 8 h without increasing until 24 h; biosorption efficiency of nickel also increased with pH changes from 2 to 7 and remained constant thereafter. The maximum sorption efficiency of nickel was obtained at 37 °C, while at 45 °C and 55 °C the nickel biosorption was reduced.

Phytoremediation has the advantage that contaminants may be treated in situ; while the major limitation is that it requires a long-term commitment, since the process is dependent on the plant ability to grow and thrive in an environment that is not always appropriate for normal plant growth.

## 9. Conclusions

Nickel is a metal of widespread distribution in the environment. Contact with soluble and insoluble nickel compounds can cause a variety of side effects on human health. Human exposure to Ni may occur through food, water or air. Workers in Ni producing and processing industries are exposed by inhalation, and to a lesser extent, dermal contact. The nervous system is one of the main target organs for Ni toxicity; in fact, it can be accumulated in the brain. Allergy to nickel and metals is caused by the materials used in our daily life; therefore, the chances of triggering the onset of allergic reactions are high. This metal can cause an allergy that manifests as contact dermatitis, headaches, gastrointestinal and respiratory manifestations. The molecular mechanisms of Ni induced neurotoxicity are still not clear, but the researchers think that oxidative stress and mitochondrial dysfunctions have a primary and important role. Mitochondrial damage induced by Ni may occur first as mitochondrial membrane potential damage, then as mitochondrial ATP concentration reduction and finally as mitochondrial DNA destruction. Damage to mitochondrial functions interferes with the mitochondrial transport chain, amplifies ROS and exacerbates oxidative stress.

In the last 25–30 years, researchers, trying to characterize the carcinogenicity, due to nickel, have uncovered that epigenetic alterations induced by nickel exposure, can perturb the epigenome. DNA hypermethylation, histone modification and interference with miRNA network, and finally condensed chromatin structure create an aberrant epigenetic landscape that contributes to nickel-induced gene silencing, tumor initiation and progression. Indeed, nickel is known to cause cancer by an epigenetic mechanism, which appears to involve the substitution of Ni^2+^ for Fe^2+^ in non-heme iron dioxygenases that are involved in DNA and histone demethylation. In vitro studies demonstrated that Ni sulphate could promote apoptosis in human epatoma cells, human T hybridoma cells human breast cancer, abrogated by curcumin. Many researchers have shown that nickel binds to amino acidic residues (as Cys, His and or Glu) of several enzymes decreasing their activity. Besides, in several enzymes the inhibitory nickel binds to an allosteric secondary site affecting activity.

Pollution of soil, water, and air, due to heavy metals, is probably the most outstanding outcome of the evolution of our society. The reclamation of soils polluted by heavy metals can be achieved with different techniques and technologies. A possible alternative to technologies is the adoption of a biological method that consists of the use of plants that may uptake and accumulate heavy metals in their tissues. The process of metal absorption and accumulation in the plant tissues is called phytoextraction, and in the case of nickel, the phytoremediation capability, which cost is not excessive, depends on the level of nickel concentration in soil.

In the intrinsic pathway, Ni^2+^ allows the Cyt C release from the mitochondria to the cytosol. Cyt C cleaves and activates caspase-9, which in turn cleaves and activates caspase-3, -6 and -7. Ni^2+^ promotes in the extrinsic pathway Fas and FasL interactions, leading to activation of caspase-8 and caspase-10 that activate caspase-3, -6 and -7. Caspase-3, -6 and -7 cleave PARP, which then induces apoptosis.

## Figures and Tables

**Table 1:** Nickel-containing foods and items and nickel toxic effects.

**Nickel Containing Foods**	Hazelnuts; cocoa and dark chocolate; fruits (almonds, dates, figs, pineapple, plums, raspberries); grains (bran, buckwheat, millet, whole grain bread, oats, brown rice, sesame seeds, sunflower seeds); seafood (shrimps, mussels, oysters, crab, salmon); vegetables (beans, savoy cabbage, leeks, lettuce, lentils, peas, spinach, cabbage), tea from drinks dispensers; soya and soya products; peanuts; licorice; baking powder.
**Nickel Containing Items**	Inexpensive jewelry; cosmetics; keys; cell phones; eyeglass frames; paper clips; orthodontic braces; stainless steel articles; nickel plated articles; clothing fasteners (zippers, snap buttons, belt buckles); electrical equipment; armaments; alloy; metallurgical and food processing industries; pigments; catalysts.
**Nickel Toxic Effects**	Contact dermatitis; headaches; gastrointestinal manifestations; respiratory manifestations; lung fibrosis; cardiovascular diseases; lung cancer; nasal cancer; epigenetic effects.

## Abbreviations

DNA	deoxyribonucleic acid;
RNA	ribonucleic acid;
AMP	adenosine 5′-monophosphate;
ATP	adenosine 5′-triphosphate;
ROS	reactive oxygen species;
SOD	superoxide dismutase;
CAT	catalase;
MDA	malondialdehyde;
NO	nitric oxide;
mRNA	messenger RNA;
Cyt C	Cytochrome C;
BAX	BCL2 Associated X;
BID	BH3 interacting domain death agonist;
Bcl-2	B-cell lymphoma-2;
SAM	S-adenosyl methionine;
Fhit	fragile histidine triad protein;
Fas	first apoptosis signal;
FasL	Fas Ligant;
NAC	N-acetylcysteine;
TEMPO	2,2,6,6-tetramethyl-1-piperidinyloxy.
